# Insecticidal activity of *Leptodactylus knudseni* and *Phyllomedusa vaillantii* crude skin secretions against the mosquitoes *Anopheles darlingi* and *Aedes aegypti*

**DOI:** 10.1186/1678-9199-20-28

**Published:** 2014-07-02

**Authors:** Frances TT Trindade, Ângela A Soares, Andréa A de Moura, Tiago B Rego, Andreimar M Soares, Rodrigo G Stábeli, Leonardo A Calderon, Alexandre de Almeida e Silva

**Affiliations:** 1 Laboratory of Insect Bioecology, Department of Biology, Federal University of Rondônia (UNIR), Porto Velho, Rondônia State, Brazil; 2 Oswaldo Cruz Foundation – Rondônia (Fiocruz – Rondônia), Porto Velho, Rondônia State, Brazil; 3 Center for the Study of Biomolecules Applicable to Health, Oswaldo Cruz Foundation – Rondônia, and Department of Medicine, Federal University of Rondônia (UNIR), Porto Velho, Rondônia State, Brazil

**Keywords:** Vector control, Anuran amphibians, Dengue, Malaria

## Abstract

**Background:**

Mosquitoes are important vectors of several diseases, including malaria and dengue, and control measures are mostly performed using chemical insecticides. Unfortunately, mosquito resistance to commonly applied insecticides is widespread. Therefore, a prospection for new molecules with insecticidal activity based on Amazon biodiversity using the anurans *Leptodactylus knudseni* and *Phyllomedusa vaillantii* was performed against the mosquito species *Anopheles darlingi* and *Aedes aegypti*.

**Methods:**

The granular secretion from anuran skin was obtained by manual stimulation, and lethal concentrations (LCs) for larvicidal and adulticidal tests were calculated using concentrations from 1-100 ppm. The skin secretions from the anuran species tested caused significant mortality within the first 24 hours on adults and larvae, but differed within the mosquito species.

**Results:**

The skin secretions from the anuran species tested caused significant mortality within the first 24 hours on adults and larvae, but differed within the mosquito species. The calculated LC_50_ of *L. knudseni* skin secretions against *An. darlingi* was 0.15 and 0.2 ppm for adults and larvae, respectively, but much higher for *Ae. aegypti*, i.e., 19 and 38 ppm, respectively. Interestingly, the calculated LCs_50_ of *P. vaillantii* against both mosquito species in adults were similar, 1.8 and 2.1 ppm, respectively, but the LC_50_ for *An. darlingi* larvae was much lower (0.4 ppm) than for *Ae aegypti* (2.1 ppm).

**Conclusions:**

The present experiments indicate that skin secretions from *L. knudseni* and *P. vaillantii* contain bioactive molecules with potent insecticide activity. The isolation and characterization of skin secretions components will provide new insights for potential insecticidal molecules.

## Background

Mosquitoes are important vectors of several diseases, including malaria and dengue fever [1]. According to the World Health Organization (WHO) [2] there were approximately 675,000 confirmed cases in 2011 of dengue fever among 19 American countries. In Brazil, most of the malaria cases occur in the northern region. Rondônia state, western Amazon, Brazil, recorded 14,510 cases in 2013, mostly transmitted by the mosquito *Anopheles darlingi*[3,4]. In 2013, of the approximately 204,650 cases of dengue fever in Brazil, 18,435 were recorded in the northern region and were transmitted by the dengue main vector, *Aedes aegypti*[5].

Vector control is mostly performed using insecticides, but, unfortunately, vector resistance is widespread among mosquitoes. Malaria mosquito resistance surveillance data from 87 countries indicated that 45 of them reported resistance to at least one insecticide used as malaria control, including pyrethroids, organophosphates and carbamates [2].

Therefore, prospection for new insecticidal molecules based on rich biodiversity sites such as the Amazon region is often performed, since microorganisms, plants and animals provide a great source of molecules for new potential drugs.

The Amazon fauna also provides the highest number of anuran species in the world and venom glands from frogs contain a variety of substances with pharmaceutical effects against tropical diseases including malaria and leishmaniasis [6,7].

*Phyllomedusa vaillantii*, a tree frog species, is often found in trees and bushes close to streams or permanent bodies of water in tropical rainforests from several countries in South America and along the Amazon basin [8]. *Phyllomedusa* skin secretion contains a rich biological mixture of peptides including antimicrobials [9,10].

*Leptodactylus knudseni*, also known as the Amazonian toad-frog, is a native frog species found in the tropical forest floor and burrows from South and Central America [11]. According to Erspamer [12], extracts from *Leptodactylus* skin were possibly used to prepare some “curares” by South American natives. The skin secretions of leptodactylids are characterized by a particular composition of amines, among them biogenic amines derivatives from imidazole, indole and phenyl-alkylamides such as leptodactyline, candicine, histamine and serotonine [13]. Besides biogenic amines, Toledo and Jared [14] also mentioned bioactive peptides such as caerulein and physalaemin in leptodactylids.

Although very few reports on the activity of anuran skin secretions on mosquitoes or other dipterans are available; some indicate that crude secretions or their components display insecticidal activity, contact toxicity and repellence [15-17]. The aim of the present study was to investigate the insecticidal activity of crude skin secretions extracted from the frogs *Leptodactylus knudseni* and *Phyllomedusa vaillantii* on the main vectors of malaria and dengue fever in Brazil, *Anopheles darlingi* and *Aedes aegypti*, respectively.

## Methods

### Animal material and crude skin secretions

*Phyllomedusa vaillantii* and *Leptodactylus knudseni* adult specimens were collected in Porto Velho, Rondônia, Brazil. Voucher specimens were identified by A. P. Lima and L. A. Calderon and deposited in the Herpetofauna Reference Collection of Rondônia (in Portuguese, Coleção de Referência da Herpetofauna de Rondônia – CRHRO) of the Federal University of Rondônia. Animals were kept inside the terrarium at the Center for the Study of Biomolecules Applicable to Health (Centro de Estudos de Biomoléculas Aplicadas à Saude – CEBio).

The granular secretion from anuran skin was obtained by manual stimulation. The dorsal glandular area of each individual was rinsed with deionized water, clarified by centrifugation, frozen, lyophilized and stored at –20°C until insecticidal assays set up.

### Mosquito collection and breeding

*Anopheles darlingi* females were collected using a modified BG BG-Sentinel™ Trap (BioQuip Products, USA) in the municipality of Candeias do Jamari, RO (8° 46′ 55″W, 63° 42′ 9″S) and sent to the Laboratory of Entomology at Fiocruz – Rondônia. *Aedes aegypti* eggs were obtained from the laboratory strain of the Laboratory of Chemical Ecology of Vector Insects (Laboratório de Ecologia Química de Insetos Vetores), UFMG, Brazil, and reared under laboratory conditions (28°C, 80% RU and 12 hour photoperiod). Then, adult mosquitoes were blood fed on rabbits and three days after, *A. darlingi* females were induced to oviposition by removing one of their wings. *Ae. aegypti* females laid eggs naturally in beaker-containing filter paper and distilled water. After hatching, the larvae were kept under laboratory conditions and fed with fish food (TetraMin® Tropical Flakes) up to 3^rd^ and 4^th^ instar, this stage being used for testing larvicides. In order to obtain adults for testing adulticide products, the same methodology was followed up to the pupal stage, when the animals were separated and transferred to larger cages.

### Insecticidal activity bioassays

The lethal concentrations (LC_50_ and LC_90_) for adult and larval mosquitoes were determined using five different concentrations (ppm: 1, 5, 10, 50, 100), each with four replicates and repeated three times on different occasions [18]. For testing larvicides, crude skin secretions of *L. knudseni* and *P. vaillanti* were diluted in water and pipetted under the surface of water in plastic cups (50 mL) containing 10 mL of distilled water and larvae (25 larvae per container) introduced in the cups 30 minutes after pipetting. For testing adulticides, crude skin secretions were diluted in 20% sucrose and pipetted on the screens of cages containing 25 mosquitoes each (30 drops of 2 μL/cage); for this mosquitoes were kept without food for 24 hours. After 30 minutes, the engorged mosquitoes were separated. The mortality of larvae and adults was recorded from 24 to 96 hours; however, the calculation of the lethal concentrations included only the 24-48 hours mortality records. The lethal concentrations (LCs) for adulticidal and larvicidal activity of skin secretions against mosquitoes were calculated using Probit analysis (Minitab, Minitab Inc). The effects of crude skin secretions on concentration and mortality for larvae and adults were analyzed by Anova on ranks (SigmaStat 2.0, 1992-1997).

## Results and discussion

**Figure 1:**
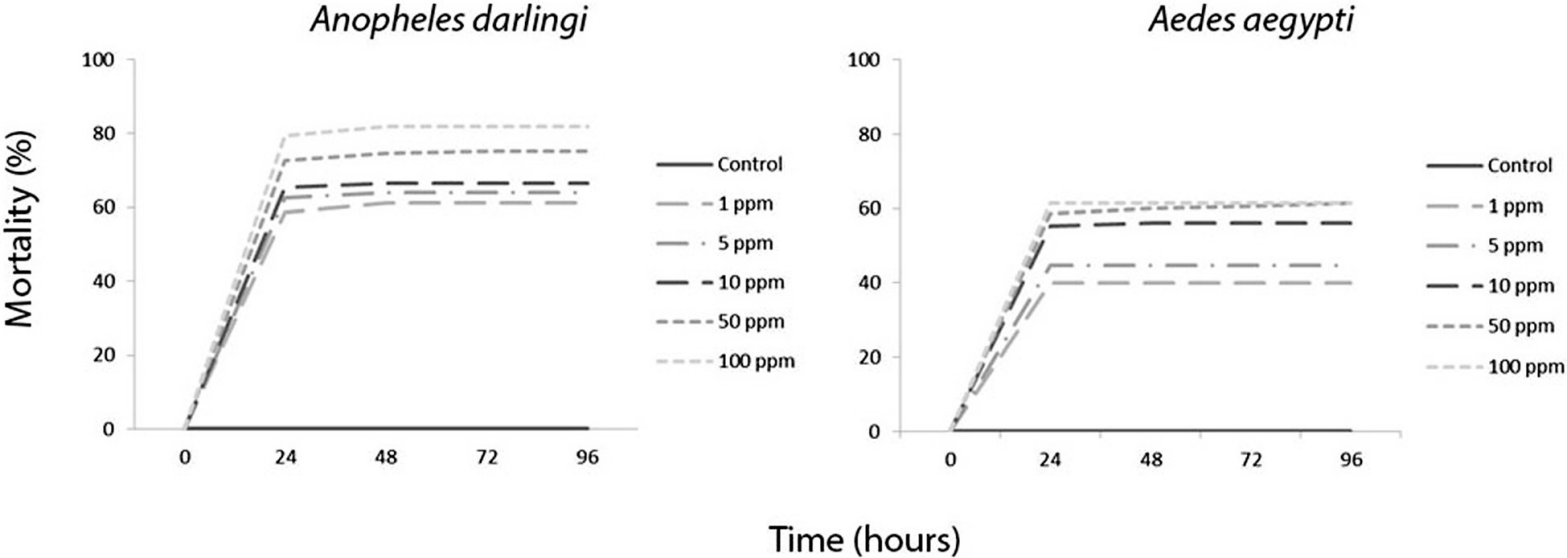
**Adulticidal activity of *Leptodactylus knudseni *** **(Anura: Leptodactylidae) crude skin secretions ingested by *Anopheles darlingi *** **and *Aedes aegypti *** **(Diptera: Culicidae) at different concentrations and time points.**

**Figure 2:**
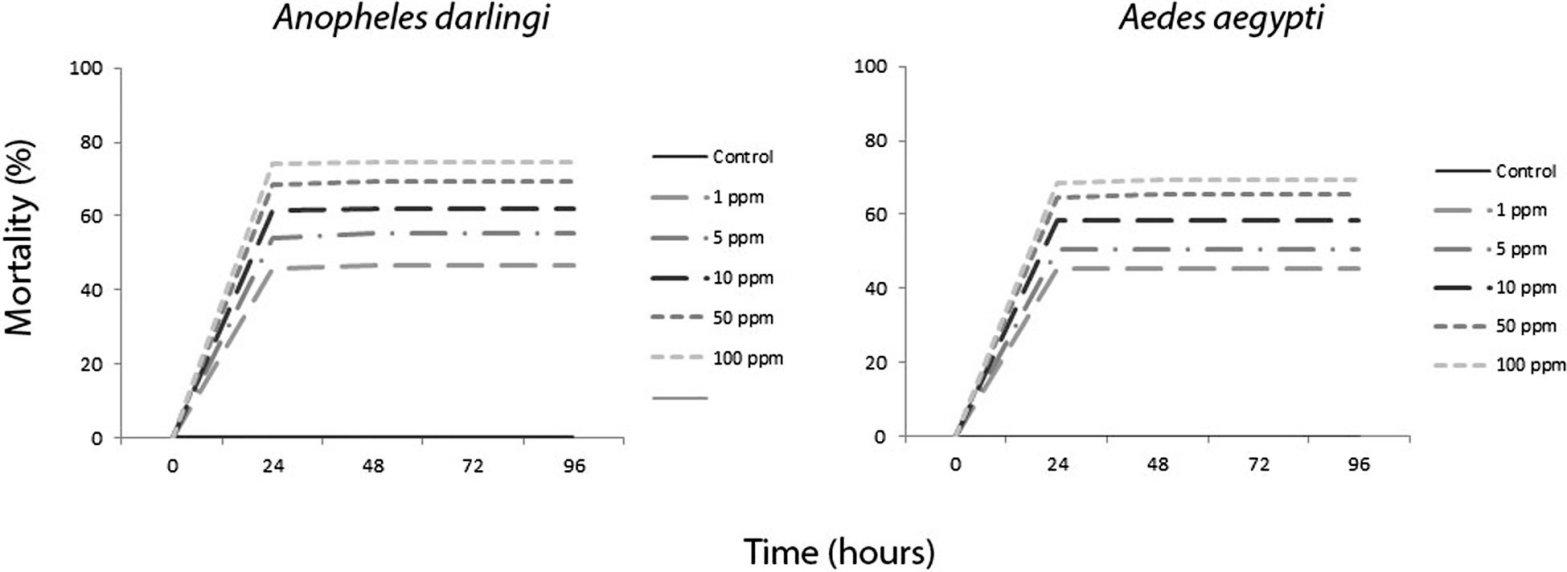
**Adulticidal activity of *Phyllomedusa vaillantii *** **(Anura: Hylidae) crude skin secretions ingested by *Anopheles darlingi *** **and *Aedes aegypti *** **(Diptera: Culicidae) at different concentrations and time points.**

**Figure 3:**
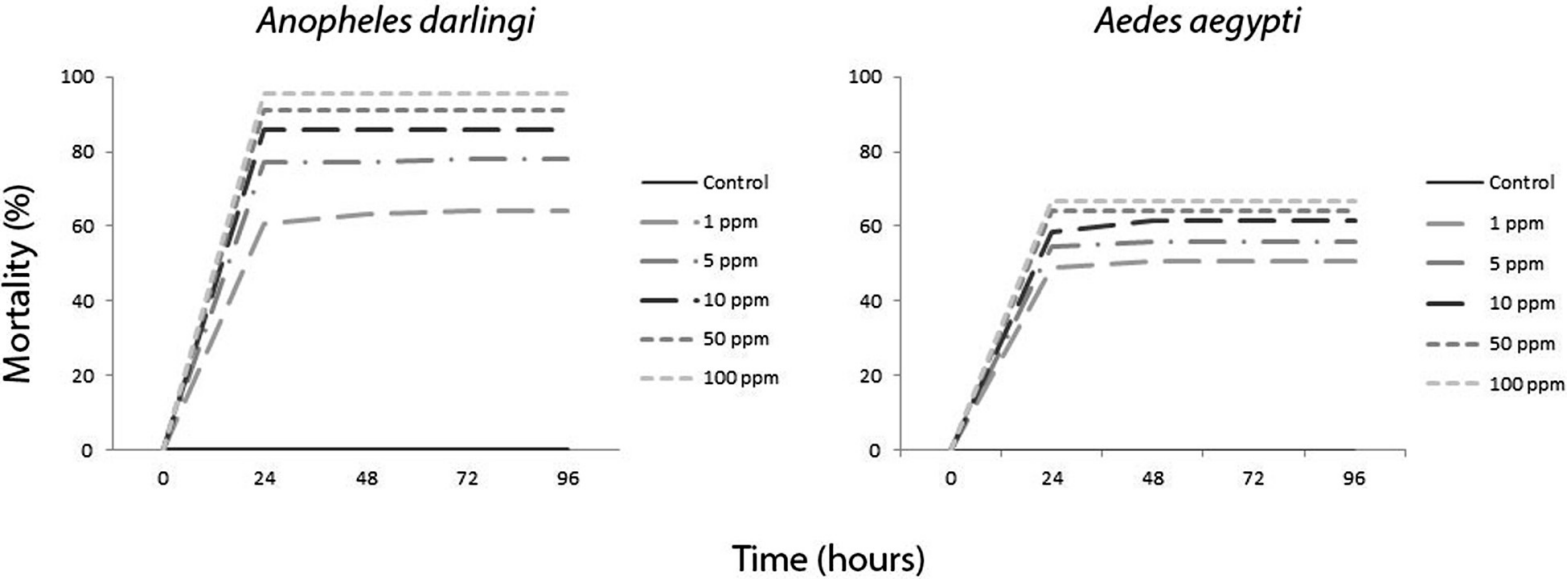
**Larvicidal activity of *Leptodactylus knudseni *** **(Anura: Leptodactylidae) crude skin secretions against *Anopheles darlingi *** **and *Aedes aegypti *** **(Diptera: Culicidae) at different concentrations and time points.**

**Figure 4:**
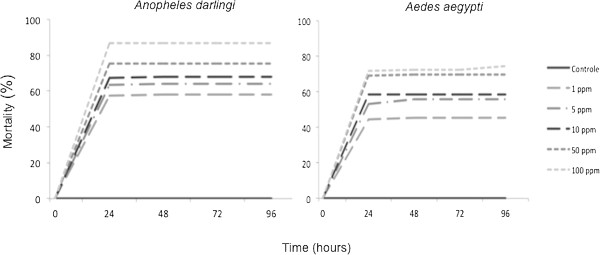
**Larvicidal activity of *Phyllomedusa vaillantii *** **(Anura: Hylidae) crude skin secretions against *Anopheles darlingi *** **and *Aedes aegypti *** **(Diptera: Culicidae) at different concentrations and time points.**

Skin secretions from the amphibian anurans *Leptodactylus knudseni* and *Phyllomedusa vaillantii* caused significant mortality (p < 0.001) on adults and larvae of the mosquitoes *An. darlingi* and *Ae. aegypti* in a concentration-dependent mortality rate. Mortality peaked in 24 hours with no significant increase afterwards (Figures 1, 2, 3 and 4)

The mortality observed for adults of *An. darlingi* and *Ae. aegypti* increased significantly after oral ingestion of 1 to 100 ppm of skin secretions from *L. knudseni* (H = 76.06, p < 0.001; H = 18.78, p < 0.001, respectively) and *P. vaillantii* (H = 77.54, p < 0.001; H = 18.72, p < 0.001 respectively).

*Anopheles darlingi* adults were more susceptible to the ingestion of *L. knudseni* skin secretions, reaching 61% of mortality with 1 ppm, while *Ae. aegypti* reached the same percentage at only with 100 ppm (Figure 1). Moreover, *An. darling* and *Ae. aegypti* had similar susceptibility to the ingestion of *P. vaillantii* skin secretions, i.e., 46% and 45% mortality at 1 ppm; 74% and 69% at 100 ppm, respectively (Figure 2). When pooled together, mortality data indicate that *An. darlingi* and *Ae. aegypti* adults were more susceptible to the skin secretions of *L. knudseni* than to *P. vaillantii* (Table 1).

**Table 1:** **Lethal concentrations (LC) in ppm for the crude skin secretions of *Leptodactylus knudseni *** **(Anura: Lepdodactylidae) and *Phyllomedusa vaillantii *** **(Anura: Hylidae) against *Anopheles darlingi *** **and *Aedes aegypti *** **(Diptera: Culicidae)**

**Anura species**	**Adult**		**Larvae**	
	***An. darlingi***	***Ae. aegypti***	***An. darlingi***	***Ae. aegypti***
	**LC**_**50**_	**LC**_**50**_	**LC**_**50**_	**LC**_**50**_
***L. knudseni***	0.15	19	0.2	38
***P. vaillantii***	1.8	2.1	0.4	2.1

PPM: parts per million, LC_50_: lethal concentration necessary to kill 50% of the larvae during assays.

Similar to adults, the larvicidal effect of anuran skin secretions on both mosquito species increased significantly with the concentration range evaluated (i.e., 1 to 100 ppm) (Figure 3). At 100 ppm, *L. knudseni* skin secretions killed 96% of *An. darlingi* (H = 77.25, p < 0.001) after 24 hours but only 66% of *Ae. aegypti* (H = 18.79, p < 0.001) at the same concentration (Figure 4). The larvae of *An. darlingi*, but not those of *Ae. aegypti*, were remarkably more susceptible to the skin secretions from *L. knudseni* and *P. vaillantii* (Table 1).

Although statistically significant, mortality differences between mosquito species at the concentrations tested decreased when larvicidal tests were performed using *P. vaillantii* skin secretions at 100 ppm, i.e. 88% and 72% for *An. darlingi* and *Ae. aegypti* (H = 76.78, p < 0.001), respectively.

Calculated lethal concentrations (LC) varied within the mosquito and anuran species tested. *Anopheles darlingi* larvae and adults presented the lowest LC_50_ (<1 ppm) for *L. Knudseni*; however, *Aedes aegypti* presented a lower LC_50_ for *P. vaillanti* skin secretions (Table 1).

Despite the lower differences in the mortality of adults and larvae of both mosquito species exposed to *P. vaillantii* skin secretions, *An. darlingi* was more susceptible to frog skin secretions tested than *Ae. aegypti* (Table 2).

**Table 2:** **General insecticidal activity (median % of mortality) effect of the crude skin secretions of *Leptodactylus knudseni *** **(Anura: Lepdodactylidae) and *Phyllomedusa vaillantii *** **(Anura: Hylidae) against *Anopheles darlingi *** **and *Aedes aegypti *** **(Diptera: Culicidae)**

**Anura species**	**Adult**		**Larvae**	
	***An. darlingi***	***Ae. aegypti***	***An. darlingi***	***Ae. aegypti***
***L. knudseni***	65.3a1	56.0b1	86.0c1	58.7d1
***P. vaillantii***	61.3a2	58.7a2	68.0b2	58.7a1

Kruskal-Wallis and Student-Newman-Keuls Tests (comparisons). Medians were calculated using mortalities from different concentrations (1-100 ppm) after 24 hours. Different numbers indicate significant (p < 0.05) differences in the same column. Different letters indicate significant (p < 0.05) differences in the same row.

Erspamer [13] argues that nearly every species of *Leptodactylus* is characterized by a particular composition of biogenic amines. In this sense, Roseghini *et al.*[19], after analyzing different alkylamines from 140 species of American frogs, stated that none of the other species studied can compete with *Leptodactylus* regarding the variety and richness of aromatic monoamines.

Biogenic amines, e.g. phenylalkylamines such as leptodactyline, have marked neuromuscular-blocking effects on mammals and LD_50_ = 235 mg/kg in mice [20].

*Phyllomedusa* species display a very rich mixture of biologically active peptides, including antimicrobial, central nervous and smooth muscle activity [] and many are known to display biological activity against important tropical diseases such as leishmaniasis and malaria parasites [6,10].

These results agree with those obtained by Weldon *et al.*[15], which reported that *Ae. aegypti* had behavioral changes upon landing after contact with toxins, such as pumiliotoxin, from dendrobatid frogs in just a few minutes after the test. Additionally, Williams *et al.*[16] reported that *Lucilia cuprina* blowflies died 4-15 minutes after tarsal contact with the skin secretion from the hylid green tree frog, *Litoria caerulea* (Anura: Hylidae) and ingestion of venom from *Litoria caerulea* (Anura: Hylidae) in a sucrose solution – 25% skin secretion (much higher concentration than used in this study) – by the blowfly *Calliphora stygia* provoked 60% mortality after 24 hours.

## Conclusion

These experiments indicate that the skin secretions from *Leptodactylus knudseni* and *Phyllomedusa vaillantii* contain bioactive molecules with potent insecticide activity. Both species belongs to anuran families that are described as rich sources of biomolecules, several of them without knowledge about their biological activity, such as hyposins from *Phyllomedusa* skin secretions. The isolation and characterization of the insecticidal molecules present in anuran skin secretions is the objective of further efforts that will be necessary in order to elucidate some aspects of the anurans and mosquito evolution, as well as their potential as source of new molecules for insecticide development.

### Ethics committee approval

The present study was approved by the Brazilian Institute of Environment and Renewable Natural Resources (IBAMA 17983-1, 27131-2, 27131-3) and Council for the Management of Genetic Resources (CGEN 010627/2011-1).

## Competing interests

The authors declare that there are no competing interests.

## Authors’ contributions

FTTT and AAS conceived this study, designed the experiments and analyzed data. ÂAS, AAM, TBR, AMS, RGS and LAC contributed to animal material collection and crude venom analysis. All authors contributed equally to the writing and revision of the article. All authors read and approved the final manuscript.
